# Traumatic Obturator Dislocation of the Hip Joint Associated with Greater Trochanter Fracture: A Case Report

**DOI:** 10.1111/os.12892

**Published:** 2021-01-28

**Authors:** Qifeng Tao, Fenglin Zhong, Chuan Wang, Hongping Wang, Chunyu Chen, Feipeng Wu, Yuping Lan

**Affiliations:** ^1^ Department of Orthopaedics Panzhihua Municipal Central Hospital Panzhihua China

**Keywords:** Dislocation, Hip fracture, Intrapelvic, Trochanter

## Abstract

**Background:**

Traumatic obturator dislocation of the hip joint associated with greater trochanter fracture is a rare injury. We used the lateral approach through the rectus abdominis to remove the femoral head dislocated into the obturator, and the posterolateral approach was used for reduction and internal fixation of the femoral greater trochanteric fracture and total hip replacement (THR). Good follow‐up results were achieved. To the best of our knowledge, this is the first report on this particular type of injury and on this approach to treating this type of injury.

**Case Report:**

The patient was hospitalized due to a traffic accident that resulted in the patient experiencing swelling and deformity accompanied by limited mobility of the left hip and left knee.

X‐ray examination and CT confirmed that the patient suffered from left hip obturator dislocation, greater trochanter fracture, pelvic fracture (Tile B), left acetabular fracture, right open tibiofibular comminuted fracture (Gustilo III), and posterior urethral injury.

The femoral head was removed from the pelvic cavity through a pararectus approach under general anesthesia. A posterolateral approach was used for open reduction, and cable internal fixation for the left intertrochanteric fracture and uncemented THR were performed.

**Results:**

The ability to work was restored 6 months after the operation. The Harris hip score, reflecting joint function, was 86 points after 2 years of follow‐up observation.

**Conclusion:**

A lateral approach of rectus abdominis to remove the dislocated femoral head in the pelvis from the obturator should be selected, along with the posterolateral approach for reduction and internal fixation of the intertrochanteric fracture and THR. This case also provides a new reference for the treatment of this type of hip fracture dislocation.

## Graphical abstract

The femoral head was removed from the pelvic cavity through a pararectus approach under general anesthesia. A posterolateral approach for open reduction and cable internal fixation for the left intertrochanteric fracture and uncemented THR were performed.

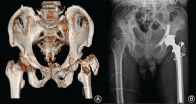

## Introduction

Due to the protection of muscles and ligaments, as well as the special anatomical structure of the hip joint, dislocation of the hip joint in adults is generally only caused by high‐energy injuries^1^, ^2^, ^3^, ^4^. According to the location of the dislocation of the femoral head, dislocation of the hip joint can be divided into anterior, central, and posterior dislocations^5^, ^6^. It should be noted that anterior dislocation can be further divided into pubic and obturator dislocations. If the energy continues to transfer, dislocation of the hip joint may occur at the same time as hip fracture^7^, ^8^. The complications of treatment of this kind of injury reported in the literature are higher^1^, ^2^, ^9^, ^10^, ^11^, ^12^, ^13^, ^14^. We diagnosed and treated a series of obturator dislocations of the hip joint associated with greater trochanter fracture. The difficulty in the treatment of this kind of disease lies in the choice of surgical method and the surgical approach through which to remove the femoral head in the obturator, because there is no guidance in the literature. We used the lateral approach of the rectus abdominis to remove the femoral head dislocated into the obturator, and the posterolateral approach was used to reduce for internal fixation of the femoral greater trochanteric fracture and total hip replacement (THR). Good follow‐up results were achieved. To the best of our knowledge, this is the first report on this particular type of injury and on this approach to treating this type of injury.

This case report was approved by the local institutional review broad. Written informed consent was obtained from the patient and his family.

### *Case Report*

A 70‐year‐old man was sitting on a chair with his left lower limb in a hip flexion, knee flexion, and abduction position when he was hit by an uncontrolled car. The patient was transported to our hospital by ambulance after primary emergency management by the ambulance medical team. When the patient arrived at the emergency department, upon further physical examination, we found swelling and tenderness in the left hip, limitation in left hip joint movement, shortening of the left lower limb by approximately 5 cm, and external rotation of approximately 80°.

**Fig. 1:**
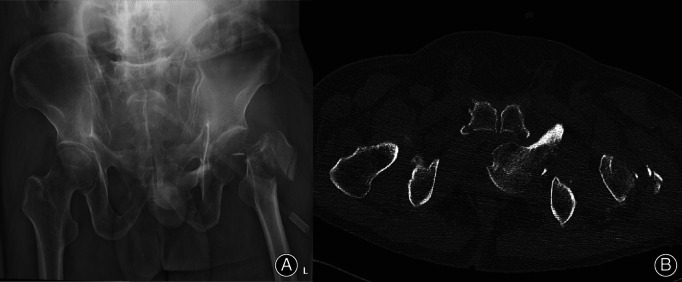
(A) Anteroposterior radiograph showing left obturator dislocation of the hip joint and greater trochanter fracture. (B) CT showing intrapelvic dislocation of the femoral head *via* the obturator.

**Fig. 2:**
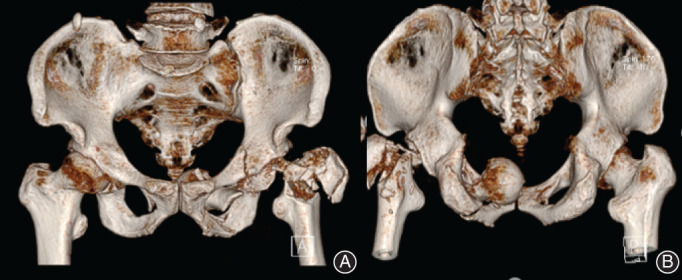
Three‐dimensional reconstruction of CT showing obturator dislocation of the hip joint with greater trochanter fracture.

Radiography and three‐dimensional CT examinations were conducted and revealed dislocation of the left hip. The femoral head had penetrated the internal structure of the obturator, and a comminuted fracture of the greater trochanteric, a fracture of the pelvis (Tile B), a fracture of the anterior column of the acetabulum (Figs 1 and 2), and a posterior urethral injury had occurred. This patient also had a tibiofibular fracture and a posterior urethral rupture.

### *Surgical Methods*

After confirming the absence of surgical contraindications, we communicated with the patient and his family and consent for surgery was obtained. Seven days after the injury, the femoral head was removed from the pelvic cavity through a pararectus approach under general anesthesia. Briefly, from the midpoint of the line between the left navel and the anterior superior iliac crest to the midpoint of the groin, a longitudinal incision of 10 cm was made. The skin and the deep subcutaneous fascia were cut, the anterior sheath of the rectus abdominis muscle was cut longitudinally, and extraperitoneal dissection was performed. The inferior epigastric artery was ligated and cut off. Protecting the spermatic cord, the iliac fascia was cut open and the iliopsoas muscle was separated from the external iliac vein and artery space. The hip and the knee were bent, and the external iliac vein and artery were pulled inward. Subperiosteal dissection exposed the anterior column and pubic branch of the acetabulum to protect the obturator nerve. The deep surface of the external iliac vessels runs through the iliac fossa. To separate the herringbone triangle, part of the attachment point of the pubic muscle was released, the anastomotic branch of the obturator artery and external iliac artery were ligated and cut off, and the obturator was stripped and exposed. There are three active bleeding points, hemostasis, and removal of the free femoral head. The acetabulum anterior column fracture block was compressed by the top bar, and the acetabulum anterior column clamp was reduced. The anterior curved titanium alloy reconstruction plate was attached to the real pelvic entrance, and the corresponding screws were inserted for fixation through drilling and sounding, while no screws were placed in the acetabulum area. The anterior sheath of rectus abdominis and the ligament of pubic tuberosity were repaired.

During the operation, it was observed that the left femoral head had entered the pelvis and extraperitoneum through the obturator foramen. There was no soft tissue connection between the femoral head and the other soft tissue. The cartilage of the femoral head was injured (Outerbridge grade IV). A posterolateral approach for open reduction and cable internal fixation (ACCORD, Smith & Nephew) for the left greater trochanteric and uncemented THR (Smith & Nephew; Echelon and Reflection; Friction interface: metal + highly crosslinked polyethylene) was performed. During the operation, fracture of the transverse acetabulum ligament, dislocation of the femoral head into the pelvis from the obturator space, and small avulsion fractures at the edge of the obturator space were observed.

### *Postoperative Functional Exercise and Follow‐Up*

**Fig. 3:**
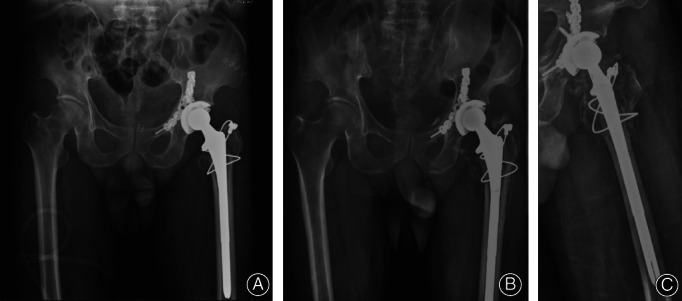
(A) Immediate radiograph after total hip replacement (THR). (B–C) Anteroposterior and lateral radiographs at the 2‐year follow‐up.

Perioperative management according to the accelerated rehabilitation management process^15^. The drugs that were used during perioperative management included enoxaparin sodium (Sanofi) 0.4 mL qd for anticoagulation and celecoxib 200 mg qd for analgesic treatment. Indomethacin was administered to prevent heterotopic ossification (HO). A postoperative X‐ray examination showed that the internal fixation of the acetabular fracture was good, the reduction and fixation of the left greater trochanteric fracture was good, and the position of the left hip joint was good. The patient started to move his hip on the bed on the second day after the operation. Urethra repair was performed 3 months after the injury. Because the patient had a serious tibiofibular fracture, partial weight‐bearing walking began 3 months after the injury, and complete weight‐bearing walking began 6 months after the injury. Follow‐up examinations showed that the fracture healed well, the hip joint prosthesis was stable, and left hip HO (Brooker grade II) was absent. HO around the left hip joint occurred 3 months after the operation (Brooker grade II). During the follow‐up period, the HO did not increase significantly, and it had no effect on the function of the left hip joint; no further treatment was needed. At the 24‐month follow‐up examination, the hip joint prosthesis was stable, no osteolytic lesions were observed, and the Harris hip score was 87.3 (Fig. 3).

## Discussion

**Fig. 4:**
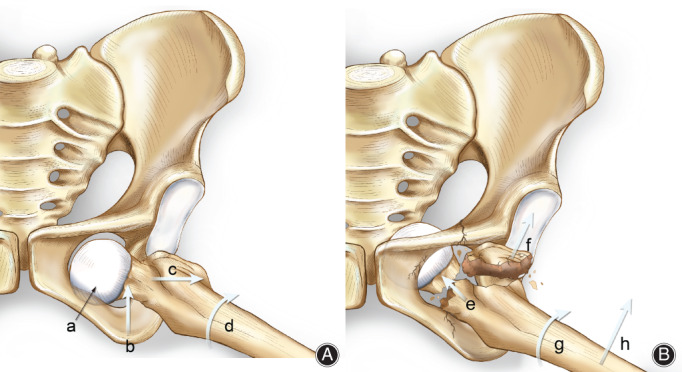
(A) Anterior dislocation of hip joint. (a) Femoral head remains in the rotation obturator position. (b) Hip flexion. (c) Femur abduction. (d) External of femur. (B) Obturator dislocation of hip joint. (e) Forces along the longitudinal direction of the femur. (f) Gluteus medius muscle traction. (g) External rotation of femur. (h) Femur abduction.

Traumatic obturator dislocation of the hip joint associated with greater trochanteric fracture is a rare type of hip dislocation. The patient was sitting in a chair when he was injured, with the left hip joint in a flexed, abducted, and externally rotated position. The external force led to dislocation of the left hip joint first, and further force led to the entry of the femoral head into the pelvis through the obturator. The greater trochanter of the femur collided with the edge of the acetabulum or the edge of the ischium foramen to form a lever, resulting in a greater trochanteric fracture. The patient also presented with ischial, acetabular, and obturator edge fractures, which increased the obturator size and created conditions for dislocation of the femoral head into the obturator. This was confirmed through intraoperative examination (Fig. 4).

The choice of open reduction and internal fixation or THR is a dilemma faced by orthopaedic surgeons. Proponents of open reduction and internal fixation believe that it can restore the bony structure of the hip joint and avoid complications associated with THR^16^. If complications such as osteonecrosis of the femoral head or traumatic arthritis occur, THR can be subsequently performed^9^, ^10^, ^17^. There have been successful cases of closed reduction and dynamic hip screw (DHS) internal fixation for the treatment of inferior dislocation of the hip joint with intertrochanteric fracture^7^. It is suggested that in patients requiring THR, there is no blood supply to the femoral head, which is often the result of injury to the femoral head, acetabular cartilage, and glenoid lip, and there is a risk of secondary fracture, nonunion, osteonecrosis of the femoral head, and osteoarthritis^2^, ^18^, ^19^, ^20^. Chang *et al*. reported a case of internal dislocation of the obturator of the right hip joint with fracture of the femoral neck that was treated with femoral head replacement. After a follow‐up duration of 10 years, the range of motion in the right hip joint was close to normal^21^. Pankaj *et al*. reported a case of THR for the treatment of an old internal dislocation of the obturator of the right hip joint. The patient was followed for 18 months and had a Harris hip score of 98^22^. During the operation, we found that the patient's femoral head was located in the obturator and out of the peritoneum. There was no soft tissue connection to the femoral head, and there were injuries to the femoral head, acetabular cartilage, and glenoid labrum. Reduction and internal fixation of the intertrochanteric fracture and THR were performed.

The posterolateral approach is often used in patients with THR complicated with a greater trochanteric fracture because the posterolateral approach can not only fully expose the hip joint but also allows a long incision during the operation. However, obturator dislocation of the hip joint occurs because the femoral head in the obturator has no bone support to provide stability of the femoral head, so it is difficult to remove the femoral head from the obturator *via* the posterolateral approach^23^. It has been reported that in two patients with internal dislocation of the obturator of the hip joint, the posterior lateral approach did not allow the removal of the dislocated femoral head from the obturator^2^, ^22^. Finally, the residual part of the femoral head was removed from the obturator^2^, ^22^. The inconsistency between the size of the femoral head and the obturator can also aggravate pelvic fractures. If the posterolateral approach is used to remove the femoral head directly from the obturator, it is very difficult to facilitate hemostasis if bleeding occurs. The vascular anatomy of the obturator is complicated. Using the lateral approach of the rectus abdominis has the following advantages. First, the anatomy of the operation level is clear, the exposure range is wide, and the operation uses longitudinal exposure, which does not cause excessive traction to the longitudinal blood vessels and nerves. Second, the external iliac vascular bundle, obturator blood vessels, and the “corona mortis” can be clearly exposed in the operation process, so that areas can be protected and dealt with quickly, and it is easy to treat the femur and stop bleeding around the head.

### *Conclusion*

We reported a rare case of traumatic obturator dislocation of the hip joint associated with greater trochanteric fracture. The lateral approach of the rectus abdominis to remove the dislocated femoral head in the pelvis from the obturator should be selected in such cases, along with the posterolateral approach for reduction and internal fixation of the intertrochanteric fracture and THR. There were no complications (e.g. hip pain or discomfort). This case also provides a new reference for the treatment of this type of hip fracture dislocation.
